# Effects of bacterial β-mannanase on apparent total tract digestibility of nutrients in various feedstuffs fed to growing pigs

**DOI:** 10.5713/ab.23.0158

**Published:** 2023-08-16

**Authors:** Ki Beom Jang, Yan Zhao, Young Ihn Kim, Tiago Pasquetti, Sung Woo Kim

**Affiliations:** 1 Department of Animal Science, North Carolina State University, Raleigh, NC 27695, USA

**Keywords:** Apparent Total Tract Digestibility, β-Mannanase, Feedstuffs, Metabolizable Energy, Pigs, Protein

## Abstract

**Objective:**

The objective of this study was to determine the effects of β-mannanase on metabolizable energy (ME) and apparent total tract digestibility (ATTD) of protein in various feedstuffs including barley, copra meal, corn, corn distillers dried grains with solubles (DDGS), palm kernel meal, sorghum, and soybean meal.

**Methods:**

A basal diet was formulated with 94.8% corn and 0.77% amino acids, minerals, and vitamins and test diets replacing corn-basal diets with barley, corn DDGS, sorghum, soybean meal, or wheat (50%, respectively) and copra meal or palm kernel meal (30%, respectively). The basal diet and test diets were evaluated by using triplicated or quadruplicated 2×2 Latin square designs consisting of 2 diets and 2 periods with a total of 54 barrows at 20.6±0.6 kg (9 wk of age). Dietary treatments were levels of β-mannanase supplementation (0 or 800 U/kg of feed). Fecal and urine samples were collected for 4 d following a 4-d adaptation period. The ME and ATTD of crude protein (CP) in feedstuffs were calculated by a difference procedure. Data were analyzed using Proc general linear model of SAS.

**Results:**

Supplementation of β-mannanase improved (p<0.05) ME of barley (10.4%), palm kernel meal (12.4%), sorghum (6.0%), and soybean meal (2.9%) fed to growing pigs. Supplementation of β-mannanase increased (p<0.05) ATTD of CP in palm kernel meal (8.8%) and tended to increase (p = 0.061) ATTD of CP in copra meal (18.0%) fed to growing pigs.

**Conclusion:**

This study indicates that various factors such as the structure and the amount of β-mannans, water binding capacity, and the level of resistant starch vary among feedstuffs and the efficacy of supplemental β-mannanase may be influenced by these factors.

## INTRODUCTION

Barley, corn, sorghum, soybean meal, and wheat are commonly used energy feeds and protein supplements in pig feeds. Due to the volatility of their economical values, some other alternative feedstuffs have been considered in feeding pigs including corn distillers dried grains with solubles (DDGS), copra meal, or palm kernel meal depending on availability in different regions [1–3]. Some of conventional and alternative feedstuffs contain high levels of non-starch polysaccharides (NSP), which are not hydrolyzed by digestive enzymes and act as anti-nutritional factors causing negative impacts on nutrient utilization in pigs [4–6]. Exogenous microbial enzymes have been considered as feed additives to improve the nutrient utilization of pigs fed with alternative feedstuffs [7–9].

Beta-mannans are complex polysaccharides that are commonly found in plant cell walls. According to Kiarie et al [10], copra meal (32.6% of total β-mannans), palm kernel meal (30.9%), and soybean meal (1.7%) are considered high sources of β-mannans compared with other feedstuffs, including barley (0.4%), corn (0.3%), corn DDGS (1.3%), sorghum (0.1%), and wheat (0.3%). Beta-mannans can also be classified in four subfamilies: linear mannan, glucomannan, galactomannan, and galactoglucomannan based on various molecules bound to β-mannan backbone such as galactose or glucose [11]. The backbone of mannan or β-glucomannan can be substituted with side chains of α-1,6-linked galactose or glucose residues. In particular, soluble β-mannans could have high viscous property and negative impacts on nutrient absorption of pigs [10]. The solubility of the β-mannans could be determined by muliple factors; i) the degree of branching, which has a positive correlation with solubility due to increased hydrophilicity with more branching, ii) the length and composition of the side chains, which can either positively or negatively impact solubility depending on their hydrophilic or hydrophobic characteristics, and iii) molecular weight, which typically has a negative correlation with solubility [12]. Beta-mannans with simple backbone structures or short side chains of hydrophobic sugars (glucose or mannose) cause increased viscosity and interfered nutrient utilization in the intestine of pigs. Although there is limited information on the characterization of specific β-mannans in the feedstuffs, the difference in β-mannan profiles among feedstuffs may cause variations in the extent of the impact on the performance and health of pigs.

Beta-mannanase has been used to hydrolyze mannans in pig feeds to improve nutrient utilization by reducing digesta viscosity, releasing nutrients for digestion [13,14], and releasing mannan-oligosaccharides which can act as prebiotics supporting the intestinal health of pigs [10,15,16]. In turn, β-mannanase could improve nutrient utilization and finally growth of pigs [10]. Efficacy of feed enzymes could vary depending on the composition of feeds due to differences in amount and structure of targeting substrates among feedstuffs [17]. It is necessary to investigate the effects of a feed enzyme on each of specific feedstuffs, which allows the estimation of its impact on feeds composed of these feedstuffs.

It is hypothesized that energy utilization and protein digestibility in various feedstuffs can be improved by the use of β-mannanase in feeds for pigs, whereas the extent of improvement can vary depending on feedstuffs composing the feed. The objective of this study was to determine the effects of β-mannanase on metabolizable energy (ME) and apparent total tract digestibility (ATTD) of protein in selected major feedstuffs including barley, copra meal, corn, corn DDGS, palm kernel meal, sorghum, and soybean meal.

## MATERIALS AND METHODS

### Animal care

The experimental protocol was approved by the Institutional Animal Care and Use Committee at North Carolina State University. The experiment was conducted at the Swine Educational Unit at North Carolina State University (Raleigh, NC, USA).

### Diets, animal, and experimental design

A basal diet was formulated with corn (94.8%) and supplemental amino acids to provide energy and essential amino acids (Table 1). Seven additional test diets were formulated by replacing a portion of the corn-basal diet with barley, corn DDGS, sorghum, soybean meal, or wheat at 50%, or with copra meal or palm kernel meal at 30%. The basal diet and test diets were evaluated by using triplicated or quadruplicated 2×2 Latin square designs consisting of 2 diets and 2 periods using a total of 54 barrows at 20.6±0.6 kg (9 wk of age). Dietary treatments were levels of β-mannanase supplementation (0 or 800 U/kg of feed). Triplicated 2×2 Latin square design was used for copra meal, corn DDGS, palm kernel meal, sorghum, and wheat with or without β-mannanase. Quadruplicated 2×2 Latin square design was used for barley, corn, and soybean meal with or without β-mannanase. Beta-mannanase (800,000 U/kg; CTCBIO Inc., Seoul, Korea) was produced from the fermentation by *Bacillus subtilis* isolate WL-7 (GenBank no. AAT27435.1) on Luria broth. The analyzed compositions of energy and nutrient composition of the feedstuffs used in this study are shown in Table 2.

### Experimental procedures and analysis

Pigs received a fixed amount of experimental diets twice daily (0800 and 1700 h) based on body weight (BW) of pigs (daily feed allowance = 0.09×BW^0.75^ kg). Pigs were weighed at the end of each period to adjust feed allowance for a subsequent period. Daily feed intake were recorded considering any feed refusal. For each phase, on d 4 at 1700 h, chromic oxide (0.3%) was added to the evening meal as an external marker to indicate the initiation of fecal collection. Sampling was done for 4-d consecutively. Fecal collection was initiated when green color from chromic oxide was visually observed in the feces after feeding a meal with chromic oxide as an indigestible maker, whereas urine sampling was initiated after the time of feeding a meal with chromic oxide. On d 8 1700 h, chromium oxide at 0.5% was added to the evening meal as an external marker for fecal collection. Fecal sampling was terminated when green color was observed in the feces in the following morning. Urine collection was terminated at the time of evening meal on d 8. Urine was collected in a plastic container with 20 mL HCl (6 *N*). The volume of urine was measured each day during the collection period, and 150 mL of the urine sample was subsampled daily. Fecal samples were weighed at the end of each day during measurement period. Urine and fecal samples were frozen (−20°C) immediately after collection.

The frozen fecal and urine samples were dried in a forced-air oven at 65°C. Daily collection of urine samples from each pig were pooled proportionally depending on the volume of urine collected on each day. Feed, urine, and fecal samples were analyzed for gross energy (GE) and crude protein (CP). Gross energy was obtained using a bomb calorimeter (Model 6300; Parr Instruments, Moline, IL, USA). Crude protein was obtained with the combustion method (method 999.03; AOAC International, 2007).

### Calculation and statistical analysis

Energy values from the excretion of GE in the feces and urine were subtracted from the intake of GE to calculate digestible energy (DE) and ME for each diet [18]. The DE and ME in barley, copra meal, corn DDGS, palm kernel meal, sorghum, soybean meal, or wheat were then calculated by a difference procedure [18,19]. The ATTD of CP was also calculated for each feedstuff by a difference procedure [18,19].

For each experiment, data were analyzed using Proc general linear model of SAS (SAS Inc., Cary, NC, USA). The experiment was based on a Latin square design, and the experimental unit was the individual pig. Period and Latin square were included as fixed effects, and pig was included as a random effect. The experimental unit was the individual pig. The statistical difference was considered significant with p<0.05, whereas 0.05≤p<0.10 was considered as tendency.

## RESULTS

Supplementation of β-mannanase did not affect GE intake in pigs fed diets containing barley, corn, corn DDGS, sorghum, or wheat (Table 3). Supplementation of β-mannanase did not affect fecal excretion of GE in pigs fed diets containing corn, corn DDGS, or wheat. Fecal excretions of GE in pigs fed diets containing barley or sorghum were reduced (p<0.05) by supplementation of β-mannanase. Supplementation of β-mannanase did not affect ATTD of GE in pigs fed diets containing corn, corn DDGS, or wheat. The ATTD of GE in the pigs fed diets containing barley or sorghum were increased (p<0.05) by supplementation of β-mannanase. Supplementation of β-mannanase did not affect DE and ME values of diets containing corn, corn DDGS, or wheat. The DE and ME values of diets containing barley were increased (p<0.05) by supplementation of β-mannanase. The DE value of diets containing sorghum tended to be increased (p = 0.052) and the ME value of the diet containing sorghum was also increased (p<0.05) by supplementation of β-mannanase.

Supplementation of β-mannanase did not affect GE intake of pigs fed diets containing copra meal, palm kernel meal, or soybean meal. Fecal excretions of GE in pigs fed diets containing copra meal (p = 0.073), palm kernel meal (p = 0.059) or soybean meal (p = 0.085) tended to be reduced by supplementation of β-mannanase. Supplementation of β-mannanase did not affect ATTD of GE in pigs fed diets containing copra meal. The ATTD of GE in the pigs fed diets containing palm kernel meal or soybean meal were increased (p<0.05) by supplementation of β-mannanase. Supplementation of β-mannanase did not affect DE and ME values of diets containing copra meal. The DE and ME values of diets containing palm kernel meal were increased (p<0.05) by supplementation of β-mannanase. The DE value of diets containing sorghum tended to be increased (p = 0.052) and the ME value of the diet was increased (p<0.05) by supplementation of β-mannanase.

Supplementation of β-mannanase did not affect CP intake and fecal excretion of CP in pigs fed diets containing barley, copra meal, corn, corn DDGS, palm kernel meal, sorghum, soybean meal, or wheat (Table 4). Supplementation of β-mannanase did not affect ATTD of CP in pigs fed diets containing barley, corn, corn DDGS, sorghum, soybean meal, or wheat. The ATTD of CP in the pigs fed diets containing copra meal tended to be increased (p = 0.061) by supplementation of β-mannanase. The ATTD of CP in the pigs fed diets containing palm kernel meal was increased (p<0.05) by supplementation of β-mannanase.

Supplementation of β-mannanase did not affect ME and ATTD of CP in corn, corn DDGS, and wheat fed to growing pigs (Table 5). Supplementation of β-mannanase improved (p<0.05) ME in barley, palm kernel meal, sorghum, and soybean meal fed to growing pigs. Supplementation of β-mannanase increased (p<0.05) ATTD of CP in palm kernel meal and tended to increase (p = 0.061) ATTD of CP in copra meal fed to growing pigs.

## DISCUSSION

Beta-mannanase is an enzyme that hydrolyzes β-mannans, complex polysaccharides in plant-based feedstuffs commonly used in swine diets, such as soybean meal, copra meal, and palm kernel meal. The addition of β-mannanase to swine feed has been shown to have several beneficial effects, including improved growth performance, nutrient digestibility, and enhanced immunity [15,20]. Supplementation of β-mannanase has been associated with reducing the negative impacts of β-mannans, which are anti-nutritional factors that can bind to nutrients and reduce its digestibility, leading to reduced growth performance in pigs [10]. It has been also known that β-mannanase breaks down these complex polysaccharides having cage effects, which physically interfere with the mode of action of endogenous or microbial enzymes to hydrolyze the nutrients inside the cell wall [21,22]. This study also shows that supplementation of β-mannanase could improve ME in barley, palm kernel meal, sorghum, and soybean meal as well as protein digestibility in copra meal and palm kernel meal. These improvement could be related to the DE of feeds containing these feedstuffs increased by supplementation of β-mannanase without affecting urinary energy. This could suggest that the β-mannanase make the nutrients more accessible for absorption, thereby contributing to an increase in ME in barley, palm kernel meal, sorghum, and soybean meal. This study can indicate the potential of β-mannanase supplementation in improving the energy utilization from barley, palm kernel meal, sorghum, and soybean meal by enhancing nutrient digestibility. Therefore, the nutrient contributions of these feedstuffs in swine feeds should be reconsidered when β-mannanase is supplemented.

In this study, the compositions of analyzed GE and CP in feedstuffs were similar except copra meal and palm kernel meal compared with the values described in NRC [23]. Comparing with previous studies, the CP in copra meal (25.7%) is slightly higher than the values (21.1%±0.7%) based on the previous reports [24–27], although the GE and CP in palm kernel meal are within the range of the values from previous studies [28–30]. According to Stein et al [31], copra meal could have variations in the nutrient composition mainly due to a differences in residual oil contents from expeller or solvent extraction.

The β-mannanase is an enzyme that breaks down β-mannans, which are contained at various levels depending on the feedstuffs [10]. Interestingly, this study shows that ME in corn, corn DDGS, wheat, or copra meal were not affected whereas ME of barley, sorghum, palm kernel meal, or soybean meal increased when β-mannanase was supplemented. The results could also indicate that the effects of β-mannanase on the ME in feedstuffs did not correspond with the β-mannan content or the ratio of soluble to total β-mannans within these feedstuffs (Table 6). Several potential reasons for these results can be speculated. The NSP compounds interact with other components in the cell wall structure of the feedstuffs. In the plant cell wall, β-mannans are typically present in the middle lamella and primary cell wall, where β-mannans contribute to the structure and rigidity [32,33]. The β-mannans interact with other polysaccharides and nutrients to form a complex that provides the cell with strength and flexibility [34–36]. If β-mannan molecules are surrounded by other NSP components, it would limit β-mannanase to physically access them, interfering with the enzymatic reaction [37,38]. Thus, the efficacy to β-mannanase would depend on physical structure of β-mannans in relation to other NSP components. In addition, the types of link and degrees of polymerization structuring β-mannans in each feedstuff vary greatly, whereas there are no clear understanding how these can influence the interaction with β-mannanase warranting further research [39].

Copra meal is rich in β-mannans, and also contains greater amounts of fat and protein as well as soluble fiber, but lower energy value compared with palm kernel meal [2]. According to Jaworski et al [40], the water binding capacity of copra meal was greater than of palm kernel meal. Water binding capacity refers to the ability of feedstuffs to hold the moisture, which is an important factor in the processing and digesting feedstuffs [41]. Soluble fiber with higher water binding capacity tend to form a viscous substance that can trap nutrients and enzymes, making them less accessible for nutrient digestion [42,43]. Feedstuffs with high water binding capacity such like copra meal can make it more difficult for digestive or exogenous enzymes to access and break down the nutrients entrapped. Previous studies showed that energy digestion of growing pigs did not response to supplementation of β-mannanase when they fed with copra meal [2,44]. This study shows that ATTD of CP in pigs fed diets containing copra meal or palm kernel meal was increased by supplementation of β-mannanase. Soluble β-mannans possessing high water binding capacity may negatively affect the protein utilization due to the hydrophilic characteristic in protein. Therefore, the results in this study indicate that there are needs to consider both the water binding capacity of feedstuffs and the enzyme activity when formulating swine feeds with exogenous enzymes.

This study shows that the ME of barley and sorghum containing relatively low levels of β-mannans was increased by the supplementation of β-mannanase. Among the feedstuffs with low NSP, Navarro et al [45] showed that barley (5.5%) and sorghum (16.2%) contain significant greater resistant starch compared with corn (1.0%) and wheat (0.4%). Resistant starch is a type of starch that is resistant to digestion in the small intestine and travels to the large intestine, where it acts as a prebiotic to promote the growth of beneficial intestinal bacteria [46]. The β-mannanase can help breaking down the NSP structure, making the resistant starch more accessible for digestion and absorption in the small intestine [47].

In addition, the ME of soybean meal was also increased by supplementation of β-mannanase. Soybean meal contains relatively high β-mannans compared with other feedstuffs such as barley, corn DDGS, corn, sorghum, and wheat [10]. Specifically, NSP can interfere the interactions among lipase, fat, and bile salt micelles in the digestive tract, ultimately leading to reduced fat digestion [48]. The study suggests that the improved energy digestibility may be due to the breakdown of β-mannans, which are primarily galactomannans, a type of soluble NSP composed of 1,4-linked β-D-mannopyranosyl residues in soybean meal [10,49,50]. The composition or types of β-mannans present in feedstuffs may influence their interactions with other nutrients or the substrate effects, potentially leading to varying responses to β-mannanase. However, supplementation of β-mannanase did not affect ATTD of CP of the pigs fed with soybean meal. One possible reason would be related to that the protein in soybean meal highly digestible for growing pigs. The results also show that soybean meal has the highest protein digestibility among the other feedstuffs. Soy protein is generally considered to be highly digestible for pigs, with a protein digestibility value of around 80% or higher [51]. Moreover, this study also shows that the changes in the ATTD of CP by supplementation of β-mannanase were detected in copra meal and palm kernel showing the lowest protein digestibility among the feedstuffs.

## CONCLUSION

Supplementation of β-mannanase improved ME in barley, palm kernel meal, sorghum, and soybean meal, and ATTD of CP in copra meal and palm kernel meal for growing pigs. This study also indicates that various factors such as the structure and the amount of β-mannans, water binding capacity, and the level of resistant starch vary among feedstuffs and thus the efficacy of supplemental β-mannanase may be influenced by these factors.

## Figures and Tables

**Table 1:** Composition of experimental diets^1)^ (%, as-fed basis)

Item	Corn basal diet
Feedstuff (%)	
Corn, yellow	94.77
L-Lys HCl	0.50
DL-Met	0.05
L-Thr	0.15
L-Trp	0.07
Salt	0.40
Monocalcium phosphorus	2.50
Limestone	1.20
Vitamin and mineral premix^2)^	0.36
Total	100.00
Calculated composition^3)^	
DM (%)	88.9
ME (Mcal/kg)	3.3
CP (%)	8.49
SID Lys (%)	0.57
Ca (%)	0.86
STTD P (%)	0.56
Mannan (%)	0.28

DM, dry matter; ME, metabolizable energy; CP, crude protein; SID, standardized ileal digestible; STTD, standardized total tract digestible.

1) Five feedstuffs including barley, corn distillers dried grains with solubles (DDGS), sorghum, soybean meal, or wheat and two feedstuffs including copra meal or palm kernel meal were replaced into the corn-basal diets at 50% and 30%, respectively, to formulate test diets with or without β-mannanase supplementation at 800 U/kg. The analyzed β-mannanase activities were 835±34 U/kg, 842±25 U/kg, 883±102 U/kg, 937±26 U/kg, 886±98 U/kg, 817±8 U/kg, 821±2 U/kg, and 906±72 U/kg in diets with barley, corn, corn DDGS, copra meal, palm kernel meal, sorghum, soybean meal and wheat, respectively.

2) The vitamin premix will supply the following per kg of complete diet: 8,433 IU of vitamin A, 1,202 IU of vitamin D_3_ as activated animal sterol, 48 IU of vitamin E, 4.0 mg of vitamin K as menadione dimethylpyrimidinol bisulfate, 6.0 mg of riboflavin, 36.2 mg of niacin, 24.1 mg of d-pantothenic acid as calcium pantothenate, 1.8 mg of folic acid, 0.24 mg of d-biotin, 0.031 mg of vitamin B_12_. The trace mineral premix will supply the following per kg of complete diet: 16.5 mg of Cu as CuSO_4_, 0.3 mg I as ethylenediamine dihydroiodide, 165 mg of Fe as FeSO_4_, 40 mg of Mn as MnSO_4_, 0.3 mg of Se as Na_2_SeO_3_, and 165 mg of Zn as ZnO.

3) Data based on Kiarie et al. [10] and NRC [23].

**Table 2:** Analyzed nutrient compositions in feedstuffs (dry matter basis)

Item	Dry matter (%)	Gross energy (kcal/kg)	Crude protein (%)	Neutral detergent fiber (%)	Acid detergent fiber (%)	Ash (%)
Barley	88.71	3,913	11.14	10.27	3.61	5.08
Copra meal	94.56	4,001	25.69	58.72	41.82	7.62
Corn	89.48	3,870	8.99	7.82	2.83	1.25
Corn DDGS	92.15	4,623	29.87	30.29	13.45	6.06
Palm kernel meal	92.22	4,494	16.34	56.87	48.82	5.46
Sorghum	89.17	3,905	9.13	10.43	4.67	1.63
Soybean meal	91.40	4,139	49.34	6.95	5.61	6.25
Wheat	89.43	3,940	13.75	10.86	3.72	2.13

Corn DDGS, corn distillers dried grains with solubles.

**Table 3:** Energy balance (dry matter basis) in feeds with various feedstuffs fed to growing pigs with or without β-mannanase

Item	β-Mannanase (U/kg of feed)^1)^		SEM	p-value

	0	800		
Barley				
GE intake (kcal)	17,276	17,342	375	0.904
GE in feces (kcal)	2,529	1,800	232	0.046
GE in urine (kcal)	260	210	22	0.133
ATTD of GE (%)	83.9	88.4	1.3	0.030
DE (diet, kcal/kg)	3,247	3,409	47	0.032
ME (diet, kcal/kg)	3,190	3,364	50	0.030
Copra meal				
GE intake (kcal)	14,535	14,420	647	0.903
GE in feces (kcal)	2,440	2,299	50	0.073
GE in urine (kcal)	199	192	29	0.874
ATTD of GE (%)	81.7	82.6	0.6	0.276
DE (kcal/kg)	3,176	3,212	24	0.332
ME (kcal/kg)	3,125	3,161	22	0.276
Corn				
GE intake (kcal)	15,033	14,868	928	0.904
GE in feces (kcal)	1,936	1,883	174	0.836
GE in urine (kcal)	171	175	22	0.907
ATTD of GE (%)	86.1	86.1	0.6	0.959
DE (kcal/kg)	3,202	3,206	19	0.895
ME (kcal/kg)	3,161	3,163	20	0.959
Corn DDGS				
GE intake (kcal/kg)	15,132	15,132	382	1.000
GE in feces (kcal/kg)	3,084	3,100	76	0.884
GE in urine (kcal/kg)	315	249	39	0.260
ATTD of GE (%)	77.5	77.9	0.4	0.552
DE (kcal/kg)	3,430	3,427	22	0.925
ME (kcal/kg)	3,341	3,357	17	0.552
Palm kernel meal				
GE intake (kcal/kg)	14,187	14,305	874	0.926
GE in feces (kcal/kg)	3,095	2,728	122	0.059
GE in urine (kcal/kg)	166	141	28	0.542
ATTD of GE (%)	76.8	79.8	0.7	0.015
DE (kcal/kg)	3,065	3,175	30	0.026
ME (kcal/kg)	3,020	3,137	28	0.015
Sorghum				
GE intake (kcal/kg)	15,695	15,809	204	0.705
GE in feces (kcal/kg)	2,130	1,756	104	0.044
GE in urine (kcal/kg)	239	229	15	0.671
ATTD of GE (%)	84.9	87.5	0.7	0.047
DE (kcal/kg)	3,324	3,420	28	0.052
ME (kcal/kg)	3,265	3,364	28	0.047
Soybean meal				
GE intake (kcal/kg)	15,074	15,074	608	1.000
GE in feces (kcal/kg)	1,926	1,747	69	0.085
GE in urine (kcal/kg)	283	245	45	0.567
ATTD of GE (%)	85.3	86.7	0.5	0.049
DE (kcal/kg)	3,329	3,377	18	0.083
ME (kcal/kg)	3,260	3,315	18	0.049
Wheat				
GE intake (kcal/kg)	12,746	12,895	722	0.887
GE in feces (kcal/kg)	1,653	1,619	69	0.737
GE in urine (kcal/kg)	206	241	35	0.490
ATTD of GE (%)	85.3	85.5	0.5	0.832
DE (kcal/kg)	3,241	3,254	19	0.627
ME (kcal/kg)	3,181	3,186	17	0.832

GE, gross energy; ATTD, apparent total tract digestibility; DE, digestible energy; ME, metabolizable energy; DDGS, distillers dried grains with solubles.

1) One unit (U) of β-mannanase activity is defined as the amount of enzyme required to releases 1 μmole of mannose reducing sugars equivalents per minute from locust bean gum (1.0%) in sodium phosphate buffer (200 mmol/L), pH 6.0 at 50°C.

**Table 4:** Apparent total tract digestibility (ATTD) of crude protein (CP) in feeds with various feedstuffs fed to growing pigs with or without β-mannanase

Item	β-mannanase (U/kg of feed)^1)^		SEM	p-value

	0	800		
Barley				
CP intake (g)	456	457	10	0.904
CP in feces (g)	107	98	4	0.190
ATTD of CP (%)	76.6	78.6	0.9	0.156
Copra meal				
CP intake (g)	519	515	23	0.902
CP in feces (g)	144	129	8	0.210
ATTD of CP (%)	72.1	75.0	1.0	0.061
Corn				
CP intake (g)	396	392	24	0.904
CP in feces (g)	88	86	5	0.864
ATTD of CP (%)	77.8	77.9	1.0	0.951
Corn DDGS				
CP intake (g)	686	686	17	1.000
CP in feces (g)	206	192	9	0.282
ATTD of CP (%)	69.9	71.9	1.2	0.247
Palm kernel meal				
CP intake (g)	402	405	25	0.926
CP in feces (g)	127	114	6	0.147
ATTD of CP (%)	68.1	71.9	1.1	0.035
Sorghum				
CP intake (g)	369	372	5	0.705
CP in feces (g)	57	24	23	0.365
ATTD of CP (%)	84.3	93.5	7	0.365
Soybean meal				
CP intake (g)	1,142	1142	46	1.000
CP in feces (g)	176	143	20.1	0.274
ATTD of CP (%)	84.9	87.5	1.4	0.219
Wheat				
CP intake (g)	387	392	22	0.887
CP in feces (g)	66	65	5.9	0.860
ATTD of CP (%)	82.8	83.6	1.1	0.635

ATTD, apparent total tract digestibility; CP, crude protein; DDGS, distillers dried grains with solubles.

1) One unit (U) of β-mannanase activity is defined as the amount of enzyme required to releases 1 μmole of mannose reducing sugars equivalents per minute from locust bean gum (1.0%) in sodium phosphate buffer (200 mmol/L), pH 6.0 at 50°C.

**Table 5:** Metabolizable energy (ME) and apparent total tract digestibility (ATTD) of crude protein in various feedstuffs fed to growing pigs with or without β-mannanase

Item	β-mannanase (U/kg of feed)^1)^		SEM	p-value

	0	800		
ME (kcal/kg)				
Barley	3,218	3,553	96	0.030
Copra meal	3,048	3,161	69	0.276
Corn	3,217	3,212	29	0.906
Corn DDGS	3,462	3,468	38	0.905
Palm kernel meal	2,751	3,091	82	0.015
Sorghum	3,262	3,457	55	0.047
Soybean meal	3,336	3,432	32	0.049
Wheat	3,197	3,207	32	0.826
ATTD of CP (%)				
Barley	70.5	74.0	1.6	0.156
Copra meal	59.3	64.5	1.8	0.061
Corn	77.8	77.9	1.0	0.952
Corn DDGS	66.3	68.9	1.5	0.248
Palm kernel meal	47.1	55.6	2.5	0.035
Sorghum	73.6	75.3	1.4	0.324
Soybean meal	84.4	87.2	1.7	0.219
Wheat	81.6	82.3	1.9	0.635

ME, metabolizable energy; DDGS, distillers dried grains with solubles; ATTD, apparent total tract digestibility; CP, crude protein.

1) One unit (U) of β-mannanase activity is defined as the amount of enzyme required to releases 1 μmole of mannose reducing sugars equivalents per minute from locust bean gum (1.0%) in sodium phosphate buffer (200 mmol/L), pH 6.0 at 50°C.

**Table 6:** Compositions of soluble and total β-mannans in feedstuffs (as-is basis)^1)^

Item	Soluble β-mannans (%)	Total β-mannans^2)^ (%)	Soluble to total β-mannans ratio
Barley	0.28	0.36	0.78
Copra meal	3.36	29.97	0.11
Corn	0.09	0.22	0.41
Corn DDGS	0.38	1.14	0.33
Palm kernel meal	4.83	28.40	0.17
Sorghum	0.11	0.09	1.22
Soybean meal	0.39	0.94	0.41
Wheat	0.18	0.27	0.67

DDGS, distillers dried grains with solubles.

1) Achieved from Kiarie et al [10].

2) Values for as-is basis were calculated based on dry matter (%) in feedstuffs reported in NRC [23].
